# Cardiac output estimation using ballistocardiography: a feasibility study in healthy subjects

**DOI:** 10.1038/s41598-024-52300-3

**Published:** 2024-01-19

**Authors:** Johannes Nordsteien Svensøy, Erik Alonso, Andoni Elola, Reidar Bjørnerheim, Johan Ræder, Elisabete Aramendi, Lars Wik

**Affiliations:** 1 Norwegian National Advisory Unit on Prehospital Emergency Medicine (NAKOS), Division of Prehospital Services, Oslo University Hospital, Oslo, Norway; 2 Institute of Clinical Medicine, Faculty of Medicine, University of Oslo, Oslo, Norway; 3 Department of Applied Mathematics, University of the Basque Country (UPV/EHU), Bilbao, Spain; 4 Department of Electronic Technology, University of the Basque Country (UPV/EHU), Eibar, Spain; 5 Division of Internal Medicine, Department of Cardiology, Ullevål Hospital, Oslo, Norway; 6 Division of Emergency Medicine, Department of Anestesiology, Ullevål Hospital, Oslo, Norway; 7 Department of Communications Engineering, University of the Basque Country (UPV/EHU), Bilbao, Spain; 8 Division of Prehospital Services, Department of Air Ambulance, Ullevål Hospital, Oslo, Norway

**Keywords:** Preclinical research, Arrhythmias, Biomarkers

## Abstract

There is no reliable automated non-invasive solution for monitoring circulation and guiding treatment in prehospital emergency medicine. Cardiac output (CO) monitoring might provide a solution, but CO monitors are not feasible/practical in the prehospital setting. Non-invasive ballistocardiography (BCG) measures heart contractility and tracks CO changes. This study analyzed the feasibility of estimating CO using morphological features extracted from BCG signals. In 20 healthy subjects ECG, carotid/abdominal BCG, and invasive arterial blood pressure based CO were recorded. BCG signals were adaptively processed to isolate the circulatory component from carotid (CCc) and abdominal (CCa) BCG. Then, 66 features were computed on a beat-to-beat basis to characterize amplitude/duration/area/length of the fluctuation in CCc and CCa. Subjects’ data were split into development set (75%) to select the best feature subset with which to build a machine learning model to estimate CO and validation set (25%) to evaluate model’s performance. The model showed a mean absolute error, percentage error and 95% limits of agreement of 0.83 L/min, 30.2% and − 2.18–1.89 L/min respectively in the validation set. BCG showed potential to reliably estimate/track CO. This method is a promising first step towards an automated, non-invasive and reliable CO estimator that may be tested in prehospital emergencies.

## Introduction

Evaluation of circulation is determinant to deliver indicated care in patients with suspected compromised circulation. The paradox is that when it is critical it is also difficult to measure. In out-of-hospital cardiac arrest (OHCA), detecting a pulse-generating rhythm (PR) that produces the return of spontaneous circulation (ROSC)^1^ is key for a prompt cessation of unnecessary chest compressions that could reinduce ventricular fibrillation^2^ and a quick initiation of post-resuscitation care^3^. Nevertheless, palpating pulse in the carotid is not a reliable measurement of circulation^4,5^ and the evaluation of 'normal breathing' proposed by current resuscitation guidelines^6^ has resulted in an unreliable indicator of circulation^7,8^. Moreover, when visually inspecting the electrocardiogram (ECG) PR can be misinterpreted as electromechanical dissociations of heart functions, namely pulseless electrical activity (PEA) and pseudo-PEA (PPEA). Both PEA and PPEA present a (quasi)-normal ECG with visible QRS complexes. During PEA there is a total loss of the contractile function of the heart, while during PPEA the heart contracts feebly, but insufficiently for a proper organ perfusion and for a recovery of consciousness^9^.

Cardiac output (CO) monitoring might be the key to differentiate PEA (nonexistent CO), PPEA (low CO) and PR (normal CO). Unfortunately, there is no ideal system for prehospital CO monitoring which should be non-invasive, easy to use, advanced skills not needed, accurate, continuous, reliable and compatible in adult/pediatric patients^10^. To date, the thermodilution technique using pulmonary artery catheter (PAC) has been considered the gold standard in CO measurement. Nevertheless, it is invasive, not feasible in the prehospital setting, and the appropriateness of using PACs in critically ill patients has been questioned since catheters are associated with increased morbidity and mortality^11,12^. For these reasons, in the last two decades a gradual transition from invasive to minimally or non-invasive techniques has been witnessed^13^. Among the minimally invasive techniques, pulse contour analysis of the arterial pressure waveform^10,14^, transpulmonary thermodilution^15^, transesophageal echocardiography^16,17^ and partial CO_2_ rebreathing^18^ are the ones implemented by manufacturers into their commercial CO monitors. However, most of these methods are not feasible for use in a prehospital emergency setting, because the equipment is either technically difficult to operate or unreliable in the prehospital scenario, time-consuming to deploy, or special skills are necessary to get reliable readings.

A suitable non-invasive and low-cost alternative is the ballistocardiography (BCG) that measures the recoil forces of the body caused by the blood pumped by the beating heart into the vascular bed^19^. The scientific literature on the use of BCG for CO estimation is scarce. The idea of estimating CO from the BCG was conceived by Starr et al. in 1940^20^. They proposed the first formula to compute stroke volume in subjects at rest using features (mainly durations and areas) from the BCG measured through a lightweight bed suspended by long cables. However, the extremely cumbersome hardware and the advent of echocardiography led the medical community to widely abandon the BCG^21^. Recently, research on BCG has reemerged thanks to the technological advancements that reduced in size the sensors. Thus, waveform features extracted from BCG sensors located on a bathroom scale^22,23^, on the seat of a chair^24^, and on body surface^25^ have been reported to track changes in CO. The amplitude of the BCG waveform recorded by sensors located on a force plate^26^ and on the wrist^27^ has been found to be positively correlated with CO. BCG acquired by sensors installed on home bathroom scale^28^, on a static-charge-sensitive bed^29^, and on the wrist^27^ has even been suggested as a surrogate measure of heart contractility. Nevertheless, none of the previous studies were conducted in a prehospital emergency setting and did not propose a method for CO estimation. Therefore, the aim of this study was to analyze the feasibility of estimating CO using morphological features extracted from BCG piezoelectric biosensors placed on the body surface of healthy subjects during test conditions that may mimic an emergency medicine situation.

## Materials

### Study setting, design and protocol

We conducted an observational quality controlled scenario based study of healthy volunteer subjects at Oslo University Hospital (Oslo, Norway). The Regional Ethics Committee for Medical and Health Research (REK 153368) and the Data Safety Commission approved the study which was registered in ClinicalTrials.gov (NCT04585568). All methods were carried out in accordance with relevant guidelines and regulations. All subjects received written and oral information about the procedures involved and signed consent before enrolment. The invasive procedure (fully described in the supplementary materials) and potential risks of radial artery cannulation was described explicitly and in detail, also as required and approved upon by the Ethical Committee (REK, see above). The study was coordinated by the Norwegian National Advisory Unit on Prehospital Emergency Medicine (NAKOS) and the Division of Prehospital Services, Oslo University Hospital.

**Figure 1:**
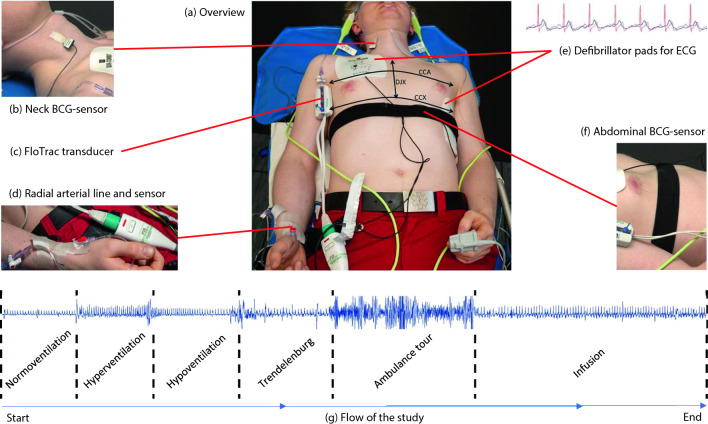
Overview of biosensor placements and study flow. (**a**) Subject placed on a stretcher. (**b**) BCG biosensor placed on the skin over the carotid artery. (**c**) FloTrac transducer for measuring arterial blood pressure. (**d**) Radial arterial line connected to the FloTrac sensor and transducer. (**e**) Defibrillation pads connected to the LIFEPAK® 15 monitor showing ECG (example of red ECG waves shown over blue BCG waves). (**f**) BCG biosensor placed on the skin over the abdominal aorta. (**g**) Flow of the study from left to right showing each phase with corresponding BCG waves. CCA, chest circumference at armpit level; CCX, chest circumference at the xiphoid process level; DJX, distance from the jugular notch to the xiphoid process.

The study was conducted in the cardiology department of the Oslo University Hospital (Ullevål Hospital) where each subject lied on an ambulance stretcher with (1) the defibrillation pads of LIFEPAK® 15 monitor/defibrillator (Stryker, Redmond, WA, USA) attached in anterolateral position for electrocardiogram (ECG, lead II) recording, (2) with the FloTrac sensor attached to the radial arterial line (see details of the procedure in the supplementary materials) and connected to the HemoSphere monitor (Edwards Lifesciences Corporation, Nyon, Switzerland) for recording the invasive arterial blood pressure that allows the CO calculation based on pulse wave contour analysis, and (3) two BCG piezoelectric biosensors (Kopera Norway AS, Stavanger, Norway) placed on the skin over the carotid artery and the abdominal aorta and connected via USB to a laptop (Lenovo ThinkPad Ultrabook) equipped with ad hoc software for recording and storing BCG signals (see Fig. 1 for an overview of biosensor placements).

The protocol consisted of six consecutive continuous phases without interruptions: Phase 1 (Normoventilation for 9 min), Phase 2 (Hyperventilation for 1 min), Phase 3 (Hypoventilation by holding the breath for 0.5 min), Phase 4 (Trendelenburg, subjects were put into a 30° Trendelenburg position for around 2 min), Phase 5 (Ambulance Tour, consisted of transportation of the subject on the stretcher three floors down in an elevator to an ambulance for a 5 min standardized ride on very bumpy roads and back to the cardiology department), and Phase 6 (Infusion, subjects immediately received a 500 ml infusion of Ringer Acetat and normoventilated for around 4 min). The graphical representation of the study protocol is shown in Fig. 1.

Monitoring and verification of the different phases were carried out using a GoPro camera attached to the stretcher. All phases’ start and endpoints were marked with annotations in addition to verbal prompts of start and stop audiotaped by the GoPro camera. After the last phase of each subject the radial arterial line was removed and pressure was applied to the insertion site for 10 min, for safety the subjects were observed by an anesthesiologist during this time. Subjects were followed up concerning pain or discomfort at the insertion site, one subject had temporary discomfort which resolved without further measures.

### Data materials

The study database contained recordings from a total of 20 healthy adult subjects. Each subject recording consisted of concurrent and continuous measurements (hereinafter signals) of the ECG, carotid and abdominal BCG (from here on BCG_c_ and BCG_a_) recorded with a sampling frequency of 250 Hz, and the CO signal at a sampling frequency of 1/2 Hz (a sample every 2 s). The CO signal was considered the ground truth and computed by the HemoSphere device through the pulse wave contour analysis of the invasive arterial blood pressure. Since each signal was recorded by a different device, all the signals were first converted to a standard open format in MATLAB (Matick, MA, USA) using our own codes, and then, an ad hoc created graphical user interface was used to concurrently visualize and time align the signals.

Recordings from all subjects were visually inspected to extract segments free of artifacts due to subject/sensor/pad movement or incorrect skin-pads contact. No segments were extracted from Hyperventilation and Ambulance Tour phases. During Hyperventilation phase the fast and abrupt way of breathing introduced movement artifacts in the signals, while vehicle movement during the Ambulance Tour drastically corrupted the BCG recordings and made analysis impossible.

The study dataset was composed of a total of 125 segments, 27/19/29/50 from Phases 1/3/4/6, containing the ECG, BCG_c_, BCG_a_ and CO signals. The mean (standard deviation, SD) duration of all the segments was 48.6 (42.3) s, while for Phases 1, 3, 4 and 6 the duration was 41.1 (27.6) s, 30.1 (7.1) s, 45.7 (28.0) s, and 61.3 (57.6) s, respectively.

## Methods

A machine learning based model to estimate the CO using morphological features extracted from BCG signals was developed in this study. The approach first preprocesses ECG, BCG_c_ and BCG_a_, then adaptively filters BCG_c_ and BCG_a_ to extract their circulatory-related components and builds a multiple linear regression model to estimate the CO using features extracted from the circulatory-related components of BCG_c_ and BCG_a_.

### Signal processing and feature computation

**Figure 2:**
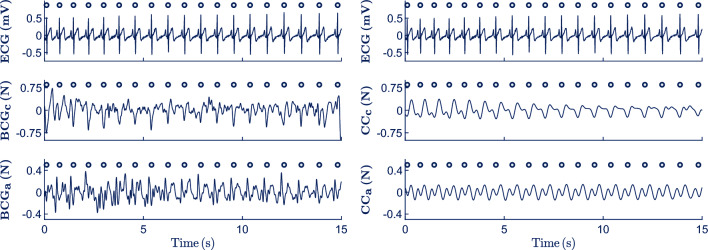
Adaptive filtering of BCG signals to extract the circulatory-related component. Preprocessed ECG, BCG_c_ and BCG_a_ are represented from top to bottom in the left panel. While preprocessed ECG and circulatory-related components of the BCG_c_ (CC_c_) and BCG_a_ (CC_a_) are depicted from top to bottom in the right panel. Dark blue circles represent the instants of the QRS complexes.

The ECG and BCG signals were first preprocessed using band-pass filters between 0.5–30 Hz and 0.5–3 Hz, respectively, to suppress the baseline drift and high frequency noise. The instants of QRS complexes were then automatically detected in the preprocessed ECG using the Hamilton-Tompkins algorithm^30^. Afterwards, BCG_c_ and BCG_a_ signals were adaptively filtered using a recursive least square (RLS) algorithm^31,32^. The RLS algorithm used the instants of QRS complexes as reference for tracking and extracting the circulatory component from BCG_c_ (CC_c_) and BCG_a_ (CC_a_) signals, respectively. Figure 2 shows the preprocessed (left) and adaptively filtered (right) signals in a 15-s segment from Phase 3 (hypoventilation). Both CC_c_ and CC_a_ reflect a fluctuation after each heartbeat. A detailed technical description of the method for estimating the circulatory component is given in the supplementary materials.

**Figure 3:**
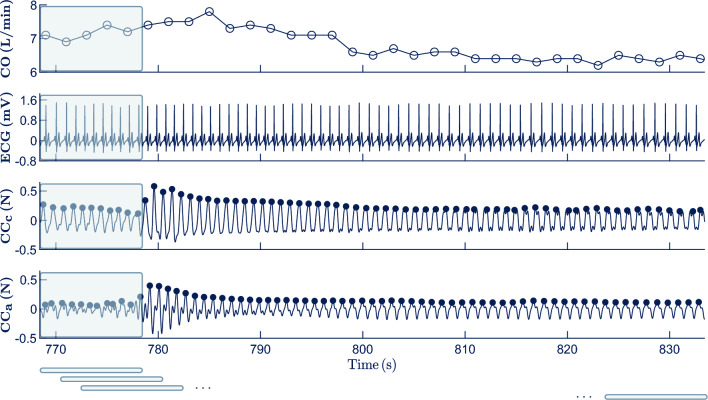
Example of a 60-s segment analyzed corresponding to Phase 4 (Trendelenburg). From top to bottom, the CO, preprocessed ECG, CC_c_ and CC_a_ are depicted. Dark blue filled dots in CC_c_ and CC_a_ represent the maxima of the fluctuations caused by each heartbeat. The large shaded rectangles in the axes illustrate the first window analyzed, whereas smaller rectangles at the bottom represent the relative position of consecutive analysis windows.

Segments were assessed using 10-s analysis windows with an 80% overlap between consecutive windows as shown in Fig. 3. A total of 66 waveform features were computed to characterize each 10-s analysis window of CC_c_ and CC_a_. Specifically, 33 features were calculated from each circulatory component. The first three features were intended to describe the statistical distribution of the circulatory component and were the standard deviation, skewness and kurtosis, respectively. The last 30 features corresponded to the median and standard deviation of 15 morphological characteristics computed on a beat-to-beat basis and with the aim of characterizing the amplitude (5), duration (4), area (3) and length (3) of the fluctuations in the circulatory component. Concretely, amplitude-related features corresponded to the peak amplitude, onset-to-peak, offset-to-peak, and the maximum and the mean between onset-to-peak and offset-to-peak amplitudes. The four duration-related features were the onset-to-peak, peak-to-offset and total durations, and the pulse width, i.e. the duration of the upper half of the fluctuation. The three features characterizing the area of the fluctuation were the onset-to-peak, peak-to-offset and total area, while length of the fluctuation was measured by computing the curve length from onset to peak, from peakt to offset, and from onset to offset (total length). The complete description of the morphological features can be found in the supplementary materials.

### Framework for the CO estimation model

Segments were randomly and patient-wise split into development (75%) and validation (25%) sets. Thus, the development/validation sets contained all the segments corresponding to the 15/5 patients randomly allocated to each set, respectively. The development set was used to select the best feature subset and to build the model based on the selected features, while the validation set was used to evaluate the performance of the model. Segments were assessed using 10-s analysis windows with an 80% overlap between consecutive windows as shown in Fig. 3. That is, for every 10-s window analyzed the median values of the 66 features were calculated and fed into the model to estimate the median CO value within the window. Thus, a total of 2471 analysis windows were divided 1939/532 into development/validation sets respectively.

Feature selection was carried out in the development set through a fivefold cross-validation (CV) scheme using a wrapping approach^33^. The baseline model was a linear regressor based on the total duration of the fluctuation in CC_c_ since this feature was the most relevant one according to the minimum redundancy maximum relevance algorithm^34^. Additional features were then added, one at a time, to the baseline model using the Plus-*l* Take-Away-*r*, PTA(*l*,*r*), algorithm^35^ with *l* = 3 and *r* = 2. Briefly, PTA(3,2) is a hybrid feature selection algorithm that at each iteration adds the most relevant feature to the *K*-feature model by stepwise including the best 3 features (a total of *K* + 3) and right after, removing stepwise the least relevant 2 features obtaining the best (*K* + 1)-feature model. The criterion to include/exclude a feature was the minimization of the mean squared error (MSE, see Eq. 2) between the CO value estimated by the model and the CO value provided by the Hemosphere (ground truth) across the fivefold composing the CV scheme. Right after selecting a feature, its squared transformation was also included in the model as long as it resulted in an MSE decrease. Features were added until no MSE decrease was obtained and therefore, the best *K*-feature subset was selected. Once features were selected, the whole development set was used to build a multiple linear regression model based on the selected best *K*-feature subset.

Finally, the validation set was used to evaluate the performance of the model in terms of (1) Bland–Altman plots and their corresponding limits for the 95% level of agreement (LOA_95%_) and (2) performance metrics such as mean absolute error (MAE), MSE and percentage of error (PE, as proposed by Critchley et al.^30^) defined by the following equations:

1 $${\text{MAE}} = \frac{1}{n}\mathop \sum \limits_{i = 1}^{n} \left| {y_{i} - \widehat{{y_{i} }}} \right|$$

2 $${\text{MSE}} = \frac{1}{n}\mathop \sum \limits_{i = 1}^{n} \left( {y_{i} - \widehat{{y_{i} }}} \right)^{2}$$

3 $${\text{PE}} = 100\frac{1.96 \sigma }{{\overline{y}}}$$

where *n* represents the number of analysis windows in the dataset, ${y}_{i}$ and $\widehat{{y}_{i}}$ are the ground truth and the model estimated CO for the *i*th analysis window respectively, $\sigma$ represents the SD of the error [real CO—estimated CO] and $\overline{y }$ depicts the mean real CO.

### Statistical analysis

Quantitative variables were expressed as mean (SD)/median (interquartile range) if they did/did not pass the Lilliefors’ normality test. Qualitative variables were expressed as absolute value and percentage, *N* (%). For comparing normal/not-normal quantitative variables by gender, the t-test/Mann–Whitney U test were performed to test for equal means/medians, respectively. A *p* value < 0.05 was considered statistically significant.

## Results

Table 1 summarizes the baseline patient characteristics of the 20 healthy subjects enrolled in the study. Subjects were predominantly female (70%) with statistically significant differences in height and chest circumference at the xiphoid process level (CCX).

**Table 1:** Baseline patient characteristics stratified by gender.

	Female	Male	Total	*p* value*
*N* (%)	14 (70%)	6 (30%)	20 (100%)	
Age^a^	24 (22–32)	27 (23–27)	25 (22–30)	0.62
Weight (kg)^a^	65 (62–76)	78 (63–83)	65 (63–80)	0.34
Height (cm)^b^	168.3 (4.8)	179.7 (5.8)	171.7 (7.3)	< 0.01
CCA (cm)^b^	91.4 (7.1)	98.0 (8.6)	93.4 (8.0)	0.14
CCX (cm)^b^	81.9 (5.9)	90.3 (6.7)	84.5 (7.2)	< 0.05
DJX (cm)	19.6 (3.1)	18.8 (2.4)	19.4 (2.8)	0.57

CCA, chest circumference at armpit level; CCX, chest circumference at the xiphoid process level, DJX, distance from the jugular notch to the xiphoid process.

*p value of the t test or Mann Whitney U test as appropriate.

^a^Expressed as median (interquartile range).

^b^Expressed as mean (SD).

**Figure 4:**
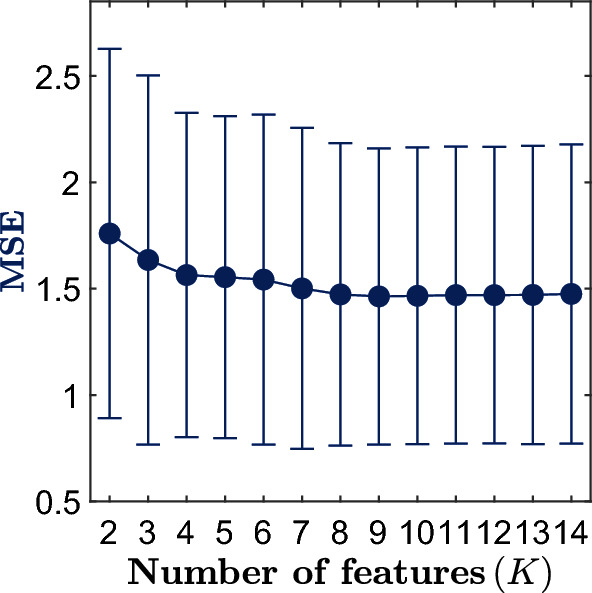
The mean squared error (MSE) in the development set as a function of the number of features included in the model.

Figure 4 represents the MSE obtained by a multiple linear regression model based on *K*-features in the fivefold CV scheme used in the development set. In other words, it shows the MSE of the model as an increasing number of features are included. There is a sharp decrease in MSE till four features are included. Then, there are slight and consecutive MSE decreases until the minimum is reached by the model containing nine features (*K* = 9).

Thus, the final regression model including the selected *K* = 9 features (details in Table 1 of the supplementary materials) was trained using the whole development set and used to produce CO estimations, $\widehat{y}$, as described in the following equation:

4 $$\begin{aligned} \hat{y} & = \beta_{0} + \beta_{1} v_{1} + \beta_{1}^{\prime } v_{1}^{2} + \beta_{2} v_{2} + \beta_{3} v_{3} + \beta_{3}^{\prime } v_{3}^{2} + \beta_{4} v_{4} + \beta_{4}^{\prime } v_{4}^{2} + \beta_{5} v_{5} + \beta_{5}^{\prime } v_{5}^{2} \\ & = 6.70 - 3.42v_{1} + 2.47v_{1}^{2} - 0.31v_{2} + 0.19v_{3} + 0.05v_{3}^{2} - 1.11v_{4} + 1.00v_{4}^{2} - 0.01v_{5} + 0.24v_{5}^{2} \\ \end{aligned}$$

where ${\beta }_{j}$ and ${\beta }_{j}{\prime}$ are the regression coefficients and ${v}_{1}-{v}_{5}$ represent the standardized (to mean/SD equal to zero/one) version of the following features: the total duration of the fluctuation in CC_c_ (${v}_{1}$), curve length from peak to offset of the fluctuation in CC_c_ (${v}_{2}$), skewness of CC_a_ (${v}_{3}$), onset to peak duration of the fluctuation in CC_a_ (${v}_{4}$), and curve length from peak to offset of the fluctuation in CC_a_ (${v}_{5}$) for the *i*th analysis window. The large magnitudes of the regression coefficients for duration related features (${v}_{1},{v}_{4}$) in both CC_c_ and CC_a_ reflect, among all features, the strongest association with the CO (*p* < 0.001). This fact is also corroborated by the existing strong negative linear correlation between the features and CO that showed a Pearson correlation coefficient of − 0.68 and − 0.38 for ${v}_{1}$ and ${v}_{4}$, respectively. We hypothesized that greater fluctuation durations might be a result of lower heart rate and consequently, lower CO.

**Figure 5:**
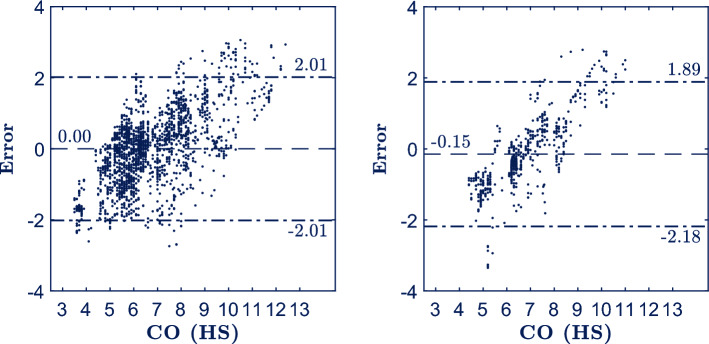
Bland–Altman plots representing the error defined as [real CO—estimated CO] as a function of the ground truth (CO computed by the HemoSphere, HS) for development (left) and validation (right) sets, respectively. Dashed line represents the mean error and dash-dotted lines represent the LOA_95%_.

Figure 5 shows the Bland–Altman plots for development (left panel, ${R}^{2}=0.39$) and validation (right panel, ${R}^{2}=0.67$) sets. The LOA_95%_ ranged from − 2.01 to 2.01 and from − 2.18 to 1.89 for development and validation sets, respectively. The model (Eq. 4) showed a MAE, MSE and PE of 0.80/0.83 L/min, 1.06/1.10 L^2^/min^2^ and 30.1%/30.2% for development/validation sets, respectively. Bland–Altman plots showed how our model slightly overestimated for CO values below 4.5 L/min and substantially underestimated for CO values above 9 L/min. For normal CO values between 4.5 and 8 L/min the error was more restrained between [− 1, 1] range. We hypothesized that these errors might be a byproduct of the optimization process of the model. The dataset of the study contained 20 healthy subjects with normal CO so that the model was trained to expect and estimate normal values (around 6.7 L/min, i,e, the intercept, ${\beta }_{0}$, of the model). Thus, when very low CO and high CO values were to be estimated the model produced estimations by subtracting and adding respectively from its baseline CO (${\beta }_{0}=6.7$ L/min), which resulted in an overestimation and underestimation.

**Figure 6:**
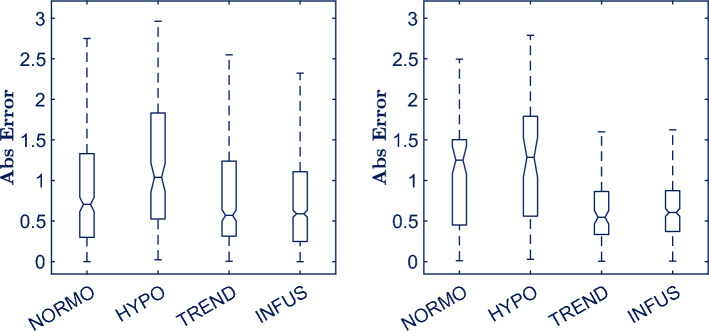
Boxplots showing the absolute error distribution across phases for development (left) and validation (right) sets, respectively.

Figure 6 illustrates the evolution of the absolute error across the different phases of the study protocol for development (left panel) and validation (right panel) sets, respectively. Similar trends can be found for both sets, although more emphasized for the development set. There is a substantial error increase from Normoventilation to Hypoventilation phase. This increase might be due to the delayed effect of the Hyperventilation phase inbetween both phases. The movement artefacts introduced by the fast and abrupt way of breathing in the Hyperventilation phase negatively affected most of the CO measurements provided by the HemoSphere monitor in the Hypoventilation phase (total duration of around 0.5 min) as the device computes CO every 2 s based on the last 20 s invasive arterial blood pressure. During Trendelenburg and Infusion phases the error decreased and stabilized. The abnormally high error presented in Normoventilation phase for the validation set compared with that obtained for the same phase in the development set is caused by the reduced number of subjects (a total of five) in the validation set and the fact that subject number one is included among those subjects. During the first phase of the first subject, i.e. the very start of the study, the control, interpretation, interiorization and implementation of the study protocol by clinicians and subjects was not optimum and resulted in lower quality of the recordings that increased the error in Normoventilation phase.

## Discussion

This is the first study comparing invasive cardiac output technology with non-invasive BCG technology in realistic scenarios of emergency medicine. Our multiple linear regression model showed promising performance estimating the CO. The PE reported was only slightly above the maximum 30% PE limit proposed by Critchley et al.^36^ for considering CO measurements interchangeable, while those PE reported by other minimally invasive or non-invasive methods were on average well above that 30% limit regardless of the ground truth used^37^. When compared against the most widely accepted gold standard (thermodilution using PAC), the pulse contour analysis based technologies and the esophageal Doppler yielded a mean (SD) PE of 41.3 (2.7)% and 42.1 (9.9)% respectively in a systematic review conducted by Peyton et al.^38^. According to the review conducted by Joosten et al.^39^ the bioimpedance, CO_2_-rebreathing, volume clamp method and pulse wave transit time showed a weighted mean PE of 42%, 40%, 56.3% and 62%, respectively. Similarly, when compared against a different reference method, namely pulse contour analysis, a weighted mean PE of 52.9%, 39.5%, 39.2% and 44.9% were reported for bioimpedance, biorereactance, volume clamp method and esophageal echoDoppler respectively^39–41^. Nevertheless, in clinical practice PE is only one metric used to evaluate methods for CO estimation, the ability to track changes in CO during challenging conditions might be as valuable in a hemodynamic unstable patient, or during cardiac arrest, as the ability to give accurate single measurements under stable conditions^38^.

The present study documents, to our knowledge, a promising first step to an accurate and reliable estimation of CO using signals that can be easily recorded in the prehospital setting. BCG biosensors are (1) very easy and fast to deploy, (2) recording and processing of BCG signals might be done either in a standalone device or even integrated into commercial monitor/defibrillators with appropriate hardware and software modifications, and (3) CO estimation would be fully automated avoiding technically difficult to operate equipment and the need for well-trained personnel to get reliable CO readings. Regarding precision, our model showed a MAE of 0.83 L/min in the validation set which represents a relative error around 12% (the mean CO of 6.7 L/min). We hypothesize that the precision of the method may be improved in future studies by first adding uncorrelated information extracted from transthoracic impedance, capnography and photoplethysmography signals recorded by the monitor/defibrillators in the prehospital setting and second using more complex, and hence less interpretable, machine learning models such as ensemble of decision trees or artificial neural networks.

This study presented some limitations. First, this was an observational quality controlled scenario based study of 20 healthy subjects. Future studies should therefore consider a larger sample size including emergency medicine patients with compromised blood flow changes to corroborate these findings. We decided not to include measurements during Hyperventilation phase. The movements required by the body to allow for following the rapid and abrupt way of breathing introduced artifacts that distorted the isolated circulatory-component and made it difficult to reliably compute the morphological features. Finally, the gold standard used was the CO measured via pulse wave contour analysis of the invasive arterial blood pressure. As stated before, the PE showed by pulse contour analysis based technologies indicated no interchangeable measures with those provided by the reference method (thermodilution). Nevertheless, pulse contour analysis has been reported to reliably track changes in CO which is key to optimize the treatment of emergency medicine patients by healthcare personnel. Ideally, thermodilution should be used. However, it is an invasive technique requiring special skills and apparatus not always achievable in the prehospital setting with the patient on an ambulance stretcher. Thus, pulse contour analysis is, to the best of our knowledge, the most suitable minimally invasive alternative to be considered as gold standard in the prehospital setting.

## Conclusion

This study presented a method to estimate the CO based only on morphological features extracted from carotid and abdominal BCG biosensors in healthy subjects. The PE and MAE showed by the method evidenced the potential of the BCG to help reliably estimate and track the CO. This method may be considered as the first step towards an automated, non-invasive, easy-to-deploy, cheap and reliable CO estimator, especially useful in prehospital emergency medicine to guide therapy, optimize treatment, and ultimately, contribute to increase survival rate.

### Supplementary Information

Supplementary Information.

## Data Availability

The data supporting the calculations, results and conclusions presented in this manuscript are not publicly available because they contain personal information but are available to any qualified researcher from co-author L.W. upon reasonable request. Correspondence should be addressed to corresponding author E.A. and requests for materials should be addressed to L.W. (lars.wik@medisin.uio.no).

## References

[1] Vincent R (2003). Resuscitation. Heart.

[2] Berdowski J, Tijssen JG, Koster RW (2010). Chest compressions cause recurrence of ventricular fibrillation after the first successful conversion by defibrillation in out-of-hospital cardiac arrest. Circ. Arrhythm. Electrophysiol..

[3] Nolan JP, Sandroni C, Böttiger BW (2021). European resuscitation council and European society of intensive care medicine guidelines 2021: Post-resuscitation care. Resuscitation.

[4] Bahr J, Klingler H, Panzer W (1997). Skills of lay people in checking the carotid pulse. Resuscitation.

[5] Tibballs J, Russell P (2009). Reliability of pulse palpation by healthcare personnel to diagnose paediatric cardiac arrest. Resuscitation.

[6] Olasveengen TM, Semeraro F, Ristagno G (2021). European Resuscitation Council Guidelines 2021: Basic Life Support. Resuscitation.

[7] Ruppert M, Reith MW, Widmann JH (1999). Checking for breathing: Evaluation of the diagnostic capability of emergency medical services personnel, physicians, medical students, and medical laypersons. Ann. Emerg. Med..

[8] Perkins GD, Stephenson B, Hulme J (2005). Birmingham assessment of breathing study (BABS). Resuscitation.

[9] Paradis NA, Martin GB, Goetting MG (1992). Aortic pressure during human cardiac arrest: Identification of pseudo-electromechanical dissociation. Chest.

[10] Mathews L, Singh KRK (2008). Cardiac output monitoring. Ann. Cardiac Anesth..

[11] Connors JAF, Speroff T, Dawson NV (1996). The effectiveness of right heart catheterization in the initial care of critically ill patients. JAMA.

[12] Sadham JD, Hull RD, Brandt RF (2003). A randomized, controlled trial of the use of pulmonary artery catheters in high-risk surgical patients. N. Engl. J. Med..

[13] Gershengorn HB, Wunsch H (2013). Understanding changes in established practice: Pulmonary artery catheter use in critically ill patients. Crit. Care Med..

[14] Hett DA, Jonas MM (2003). Non-invasive cardiac output monitoring. Intensive Crit. Care Nurs..

[15] Sakka SG, Reuter DA (2012). The transpulmonary thermodilution technique. J. Clin. Monit. Comput..

[16] Poelaert J, Schmidt C, Colardyn F (1998). Transoesophageal echocardiography in the critically ill. Anaesthesia.

[17] Poelaert J, Schmidt C, Van Aken H (1999). A comparison of transoesophageal echocardiographic Doppler across the aortic valve and the thermodilution for estimating cardiac output. Anaesthesia.

[18] Jaffe MB (1999). Partial CO_2_ rebreathing cardiac output–operating principles of the NICO system. J. Clin. Monit. Comput..

[19] Inan OT, Migeotte PF, Park KS (2015). Ballistocardiography and seismocardiography: A review of recent advances. IEEE J. Biomed. Health Inform..

[20] Starr I, Schroeder HA (1940). Ballistocardiogram. II. Normal standards, abnormalities commonly found in diseases of the heart and circulation, and their significance. J. Clin. Invest..

[21] Giovangrandi, L. *et al.* Ballistocardiography—A method worth revisiting. in *Annual International Conference of the IEEE Engineering in Medicine and Biology Society*, Vol. 2011. 4279–4282 (2011). 10.1109/IEMBS.2011.6091062 PMC4274997 22255285

[22] Inan OT, Etemadi M, Paloma A (2009). Non-invasive cardiac output trending during exercise recovery on a bathroom-scale-based ballistocardiograph. Physiol. Meas..

[23] Yazdi D, Sridaran S, Smith S (2021). Noninvasive scale measurement of stroke volume and cardiac output compared with the direct fick method: A feasibility study. J. Am. Heart Assoc..

[24] Kurumaddali, B. *et al.* Cardiac output measurement using ballistocardiogram. in *The 15th International Conference on Biomedical Engineering* 861–864 (Springer, 2014).

[25] Hossein A, Mirica DC, Rabineau J (2019). Accurate detection of dobutamine-induced haemodynamic changes by kino-cardiography: A randomised double-blind placebo-controlled validation study. Sci. Rep..

[26] Van Rooij, B. J. *et al.* Non-invasive estimation of cardiovascular parameters using ballistocardiography. In *Annual International Conference of the IEEE Engineering in Medicine and Biology Society* 1247–1250 (2015). 10.1109/EMBC.2015.7318593 26736493

[27] Steffensen TL, Schjerven FE, Flade HM (2023). Wrist ballistocardiography and invasively recorded blood pressure in healthy volunteers during reclining bike exercise. Front. Physiol..

[28] Etemadi M, Inan OT, Giovangrandi L (2011). Rapid assessment of cardiac contractility on a home bathroom scale. IEEE Trans. Inf. Technol. Biomed..

[29] Lindqvist A, Pihlajamäki K, Jalonen J (1996). Static-charge-sensitive bed ballistocardiography in cardiovascular monitoring. Clin. Physiol..

[30] Hamilton PS, Tompkins WJ (1986). Quantitative investigation of QRS detection rules using the MIT/BIH arrhythmia database. IEEE Trans. Biomed. Eng..

[31] Alonso E, Aramendi E, Daya M (2016). Circulation detection using the electrocardiogram and the thoracic impedance acquired by defibrillation pads. Resuscitation.

[32] Alonso E, Irusta U, Aramendi E (2020). A machine learning framework for pulse detection during out-of-hospital cardiac arrest. IEEE Access.

[33] James G, Witten D, Hastie T (2013). An Introduction To Statistical Learning: With Applications in R.

[34] Ding C, Peng H (2005). Minimum redundancy feature selection from microarray gene expression data. J. Bioinform. Comput. Biol..

[35] Stearns, S. D. On selecting features for pattern classifiers. In *Proceedings of 3rd International Conference on Pattern Recognition* 71–75 (1976).

[36] Critchley LAH, Critchley JAJH (1999). A meta-analysis of studies using bias and precision statistics to compare cardiac output measurement techniques. J. Clin. Monit. Comput..

[37] Scheeren TWL, Ramsay MAE (2019). New developments in hemodynamic monitoring. J. Cardiothorac. Vasc. Anesth..

[38] Peyton PJ, Chong SW (2010). Minimally invasive measurement of cardiac output during surgery and critical care: A meta-analysis of accuracy and precision. Anesthesiology.

[39] Joosten A, Desebbe O, Suehiro K (2017). Accuracy and precision of non-invasive cardiac output monitoring devices in perioperative medicine: A systematic review and meta-analysis. Br. J. Anaesth..

[40] McLean AS, Huang SJ, Kot M (2011). Comparison of cardiac output measurements in critically ill patients: FloTrac/Vigileo vs transthoracic Doppler echocardiography. Anaesth. Intensive Care.

[41] Van der Spoel AG, Voogel AJ, Folkers A (2012). Comparison of noninvasive continuous arterial waveform analysis (Nexfin) with transthoracic Doppler echocardiography for monitoring of cardiac output. J. Clin. Anesth..

