# Supplementary material for: Cardiac output estimation using ballistocardiography: a feasibility study in healthy subjects
Source: Sci Rep. 2024 Jan 19;14:1671. doi: 10.1038/s41598-024-52300-3 (PMC10796317; doi:10.1038/s41598-024-52300-3)
Supplement: Supplementary file 1 — Supplementary Information. [file 41598_2024_52300_MOESM1_ESM.pdf]

Cardiac output estimation using ballistocardiography - A feasibility study in healthy subjects

Johannes Nordsteien Svensøy[1,2,&], Erik Alonso[3,&,*], Andoni Elola[4], Reidar Bjørnerheim[5], Johan Ræder[2,6], Elisabete Aramendi[7], Lars Wik[1,8]

[1]Norwegian National Advisory Unit on Prehospital Emergency Medicine (NAKOS), Division of Prehospital Services, Oslo University Hospital, Oslo, Norway

[2]Institute of Clinical Medicine, Faculty of Medicine, University of Oslo, Oslo, Norway.

[3]Department of Applied Mathematics, University of the Basque Country UPV/EHU, Bilbao, Spain.

[4]Department of Electronic Technology, University of the Basque Country (UPV/EHU), Eibar, Spain.

[5]Division of Internal Medicine, Department of Cardiology, Ullevål Hospital, Oslo, Norway.

[6]Division of Emergency Medicine, Department of Anestesiology, Ullevål Hospital, Oslo, Norway.

[7]Department of Communications Engineering, University of the Basque Country (UPV/EHU), Bilbao, Spain.

[8]Division of Prehospital Services, Department of Air Ambulance, Ullevål Hospital, Oslo, Norway.

[&]**Johannes N. Svensøy and Erik Alonso are joint first authors**

[*]**Corresponding author:** Erik Alonso (erik.alonso@ehu.eus)

Supplementary materials

Invasive procedure and safety

The invasive procedure conducted in the study consisted in the insertion of a radial arterial line catheter. This procedure was necessary for using the pulse contour waveform technology that measures the blood pressures to calculate stroke volume and cardiac output when doppler echocardiography was impossible to do. There was a need of a technology that can be used out of hospital in emergency medicine cases where doppler echocardiography is impossible to use.

To perform the invasive procedure, volunteers were met and taken care of by a dedicated safety nurse when arriving at the hospital and throughout of the study. The safety nurse put on EMLA cream (topical anaesthetic) on the skin over the radial artery 30 minutes

before canulation. A consultant in anesthesiology using an ultrasound (US) image inserted an arterial line catheter according to the hospital's standard for arterial access to the artery under sterile conditions for each volunteer at the pre-operative area (entrance to the operating theater). After the artery lumen was "hit" (verified by US visualization and backflow of blood in the needle) but not penetrated, the catheter was flushed and secured. The volunteer waited 15 minutes to verify no side effects before followed by the safety nurse to the study area in the cardiology department. Trained personnel connected HemoSphere FloTrac blood pressure sensor (Edwards Lifesciences Corporation, Nyon, Switzerland) to the artery line for continuous monitoring. The volunteers were informed to verbally report any discomforts. After each volunteer had been through the study protocol the arterial line was removed by applying pressure to the insertion site for 10 minutes while observed for bleeding or discomforts and a pressure bandage was applied. Volunteers were followed up concerning pain or discomfort for 30 minutes. All volunteers could call a mobile phone number 24/7 if they had any concerns or questions after the study day.

Adaptive extraction of the circulatory component

Healthy subjects present a pulse so that the ballistocardiogram (BCG) signal, $s_{\mathrm{bcg}}(n)$, can be expressed using the following additive model:

$$s_{\mathrm{bcg}}(n) = s_{\mathrm{rest}}(n) + CC(n) \tag{1}$$

where sample index n is related to time by $t = n/f_s$, with a sampling frequency of $f_s = 250\,\mathrm{Hz}$. $s_{\mathrm{rest}}(n)$ contains the BCG baseline value and artifacts mostly due to sensor movement and skin-sensor contact. Each effective contraction of the heart ejects blood into the aorta and produces fluctuations in the BCG, that is the circulatory-related component, $CC(n)$. Consecutive fluctuations slightly vary in amplitude and duration. Hence, $CC(n)$ presents a quasi-periodic nature and can be modeled using a Fourier series with N terms of slowly time-varying amplitudes and frequencies:

$$CC(n) = \sum_{k=1}^{N} a_k(n)\cos(k\,\omega_0(n)\,n) + b_k(n)\sin(k\,\omega_0(n)\,n) \tag{2}$$

where N represents the number of harmonics. The Fourier coefficients $a_k(n)$ and $b_k(n)$ represent the in-phase and quadrature amplitudes of the model, and $\omega_0(n) = 2\pi f_0(n)/f_s$ its fundamental frequency. The sample indices of the R peaks of the QRS complexes, r_i, were detected using the Hamilton-Tompkins algorithm [1, 2] and used to compute $f_0(n)$ as follows:

$$f_0(n) = \frac{f_s}{r_{i+1} - r_i} \quad \forall n \in (r_i, r_{i+1}] \tag{3}$$

where $f_0(n)$ is constant within each QRS interval, but varies for consecutive intervals.

The estimation of $a_k(n)$ and $b_k(n)$ was carried out through the Recursive Least Squares (RLS) adaptive algorithm [3, 4, 5]. The RLS algorithm was used with the classical configuration for adaptive interference canceling proposed by Widrow et al. [6] illustrated in Figure 2.

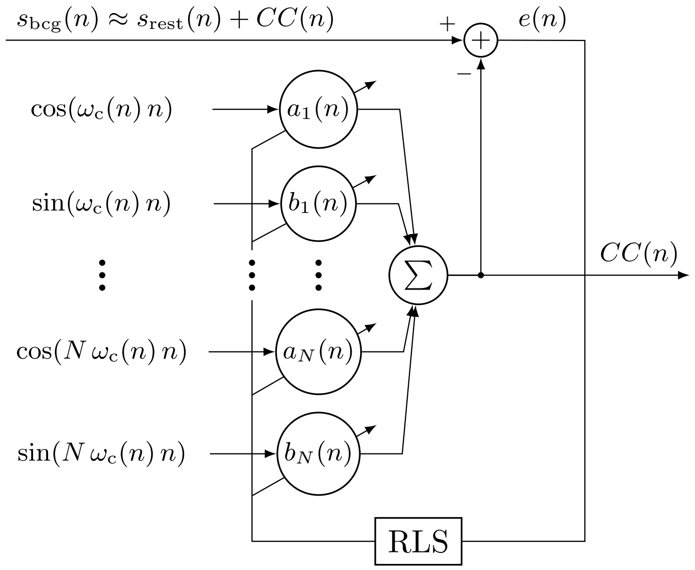

Figure 1. Block diagram of the RLS adaptive filter used to extract the circulatory component, $CC(n)$.

Using matrix notation, $CC(n) = \mathbf{x}^T(n)\mathbf{w}(n)$ where

$$\mathbf{x}(n) = \begin{pmatrix} \cos(\omega_0(n)\, n) \\ \sin(\omega_0(n)\, n) \\ \vdots \\ \cos(N\, \omega_0(n)\, n) \\ \sin(N\, \omega_0(n)\, n) \end{pmatrix} = \begin{pmatrix} x_1(n) \\ x_2(n) \\ \vdots \\ x_{2N-1}(n) \\ x_{2N}(n) \end{pmatrix}, \tag{4}$$

$$\mathbf{w}(n) = \begin{pmatrix} a_1(n) \\ b_1(n) \\ \vdots \\ a_N(n) \\ b_N(n) \end{pmatrix} = \begin{pmatrix} w_1(n) \\ w_2(n) \\ \vdots \\ w_{2N-1}(n) \\ w_{2N}(n) \end{pmatrix}, \tag{5}$$

are the so-called reference signal (harmonics) and weight vector (coefficients), respectively. The RLS algorithm searches for the $\mathbf{w}(n)$ that minimizes the following cost function

$$C(n) = \sum_{i=1}^{n} \lambda^{n-i} |e(i)|^2 \qquad 0 < \lambda \leq 1 \tag{6}$$

that represents a weighted squared error where the error, $e(n)$, is the difference between the desired signal, $d(n) = s_{\mathrm{bcg}}(n)$, and the estimated circulatory component, $CC(n)$:

$$e(n) = s_{\mathrm{bcg}}(n) - CC(n) = s_{\mathrm{bcg}}(n) - \mathbf{x}^T(n)\mathbf{w}(n) \tag{7}$$

and λ is the so-called forgetting factor that governs the convergence rate and stability of the RLS algorithm. A small λ speeds up the convergence rate at the risk of turning the process unstable [4, 5, 7]. The minimization of $C(n)$ results in the following update equations for the $\mathbf{w}(n)$:

$$\mathbf{F}(n) = \frac{1}{\lambda}\left[\mathbf{F}(n-1) - \frac{\mathbf{F}(n-1)\mathbf{x}(n)\mathbf{x}^T(n)\mathbf{F}(n-1)}{\lambda + \mathbf{x}^T(n)\mathbf{F}(n-1)\mathbf{x}(n)}\right] \tag{8}$$

$$\mathbf{w}(n) = \mathbf{w}(n-1) + \mathbf{F}(n)\mathbf{x}(n)e(n) \tag{9}$$

where the gain matrix $\mathbf{F}(n)$ and the weight vector are initialized to $\mathbf{F}(0) = 0.03\mathbf{I}_{2N}$ and $\mathbf{w}(0) = \mathbf{0}^T$ with $\mathbf{I}_{2N}$ representing the identity matrix of order $2N$.

The optimization of the configuration of the RLS algorithm consists in tuning two parameters N and λ. In this study, $N = 4$ and $\lambda = 0.9993$ were selected based on our previous experiences extracting circulatory-related components [3, 7] and optimization results obtained using the development set.

Feature extraction

A total of 66 waveform features were computed to characterize the amplitude, duration, area and length of the fluctuations for each 10-s analysis window of the carotid, $CC_c(n)$, and abdominal, $CC_a(n)$, circulatory components. Specifically, 33 features were calculated from each circulatory component. The first three features were the standard deviation, skewness and kurtosis of the circulatory component aiming to describe its statistical distribution. The last 30 features corresponded to the median and standard deviation of the following 15 morphological characteristics were computed on a beat-to-beat basis:

1. **Peak amplitude (a_M):** the amplitude of the highest peak in the fluctuation as shown in Fig. 2.

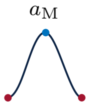

Figure 2. a_M computation.

2. **Onset-peak amplitude (A_1):** the peak-to-trough amplitude from onset to peak of the fluctuation (see Fig. 3).

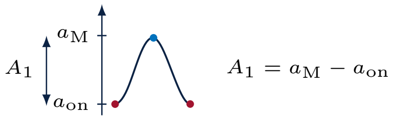

Figure 3. A_1 computation.

3. **Offset-peak amplitude (A_2):** the peak-to-trough amplitude from offset to peak of the fluctuation (see Fig. 4).

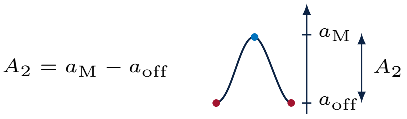

Figure 4. A_2 computation.

4. **Maximum amplitude (A_max):** the maximum amplitude between A_1 and A_2.
5. **Mean amplitude (A_mean):** the mean value of amplitudes A_1 and A_2.
6. **Onset-peak duration (D_1):** the duration from the onset to the peak of the fluctuation as illustrated in Fig. 5.

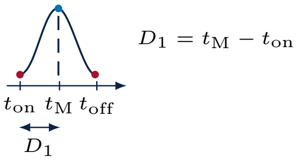

Figure 5. Calculation of D_1.

7. **Peak-offset duration (D_2):** the duration from the peak to the offset of the fluctuation as shown in Fig. 6.

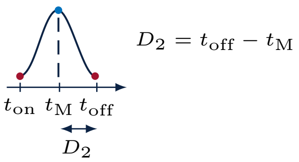

Figure 6. Calculation of D_2.

8. **Total duration (D_t):** the duration from the onset to the offset of the fluctuation (see Fig. 7).

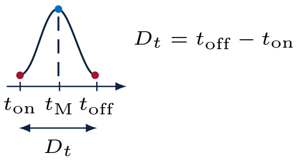

Figure 7. Calculation of D_t.

9. **Pulse width (P_{w}):** the duration of the upper half of fluctuation (see Fig. 8).

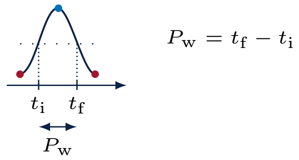

Figure 8. Calculation of D_t.

10. **Onset-peak area (Ar_1):** the area from the onset to the peak of the fluctuation as illustrated in Fig. 9 where $T_s = 1/f_s$ represents the sampling period, the inverse of the sampling frequency, f_s.

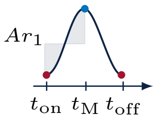

$$Ar_1 = \sum_n \frac{|CC(n)|+|CC(n+1)|}{2} T_s \quad t_{\text{on}} \leq nT_s < t_{\text{M}}$$

Figure 9. Computation of Ar_1.

11. **Peak-offset area (Ar_2):** the area from the peak to the offset of the fluctuation as shown in Fig. 10.

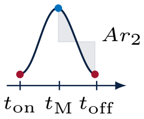

$$Ar_2 = \sum_n \frac{|CC(n)|+|CC(n+1)|}{2} T_s \quad t_{\text{M}} \leq nT_s < t_{\text{off}}$$

Figure 10. Computation of Ar_2.

12. **Total area (Ar_t):** the area from the onset to the offset of the fluctuation (see Fig. 11).

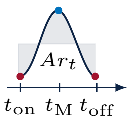

$$Ar_2 = \sum_n \frac{|CC(n)|+|CC(n+1)|}{2} T_s \quad t_{\mathrm{on}} \leq nT_s < t_{\mathrm{off}}$$

Figure 11. Computation of Ar_t.

13. **Onset-peak length (L_1):** the curve length from the onset to the peak of the fluctuation as illustrated in Fig. 12.

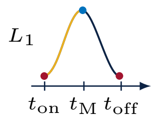

$$L_1 = \sum_n \sqrt{(T_s)^2 + (CC(n+1) - CC(n))^2} \quad t_{\mathrm{on}} \leq nT_s < t_{\mathrm{M}}$$

Figure 12. Calculation of L_1.

14. **Peak-offset length (L_2):** the curve length from the peak to the offset of the fluctuation as shown in Fig. 13.

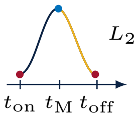

$$L_2 = \sum_n \sqrt{(T_s)^2 + (CC(n+1) - CC(n))^2} \quad t_{\mathrm{M}} \leq nT_s < t_{\mathrm{off}}$$

Figure 13. Calculation of L_2.

15. **Total length (L_t):** the curve length from the onset to the offset of the fluctuation (see Fig. 14).

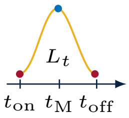

$$L_t = \sum_n \sqrt{(T_s)^2 + (CC(n+1) - CC(n))^2} \quad t_{\mathrm{on}} \leq nT_s < t_{\mathrm{off}}$$

Figure 14. Calculation of L_t.

Multiple linear regression model

All the details of the final multiple linear regression model are shown in Table 1.

Table 1. Details of the adjusted multiple linear regression model.

Variable	Estimate	SE	95% CI	t-statistic	p-value
Intercept	6.70	0.023	[6.65, 6.75]	286.55	0
v_1	-3.42	0.213	[-3.84, -3.00]	-15.99	<0.001
v_1^2	2.47	0.215	[2.05, 2.89]	11.46	<0.001
v_2	-0.31	0.026	[-0.36, -0.26]	-11.99	<0.001
v_3	0.19	0.026	[0.14, 0.24]	7.16	<0.001
v_3^2	0.05	0.025	[0.00, 0.10]	1.91	0.056
v_4	-1.11	0.120	[-1.35, -0.87]	-9.26	<0.001
v_4^2	1.00	0.123	[0.76, 1.24]	8.19	<0.001
v_5	-0.01	0.069	[-0.15, 0.13]	-0.22	0.828
v_5^2	0.24	0.068	[0.11, 0.37]	3.54	<0.001

Abbreviations: **SE** standard error, **CI** confidence interval.

Additional results

Figure 15 shows boxplots representing the evolution of the absolute error across the different phases of the study protocol for development (top panels) and validation (bottom panel) sets, respectively.

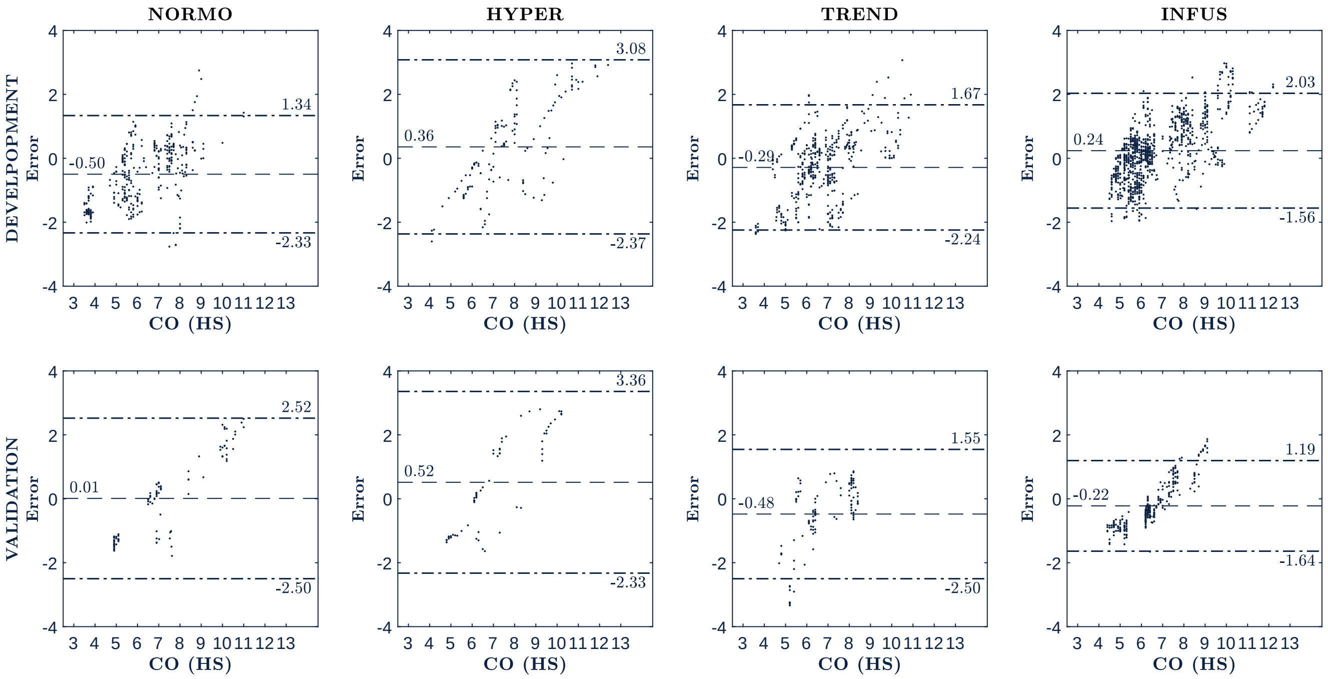

Figure 2. Bland-Altman plots for each phase in the development (top) and validation (bottom) sets.

References

[1] J. Pan et al. *A real-time QRS detection algorithm.* IEEE Trans Biomed Eng 1985;32:230-236.

[2] P. S. Hamilton et al. *Quantitative investigation of QRS detection rules using the MIT/BIH arrhythmia database.* IEEE Trans Biomed Eng 1986;33:1157-1165.

[3] E. Alonso et al. *Circulation detection using the electrocardiogram and the thoracic impedance acquired by defibrillation pads.* Resuscitation 2016;99:56-62.

[4] I. Isasi et al. *A machine learning shock decision algorithm for use during piston-driven chest compressions.* IEEE Trans Biomed Eng 2019;66(6):1752-1760.

[5] I. Isasi et al. *Automatic Cardiac Rhythm Classification With Concurrent Manual Chest Compressions.* IEEE Access 2019;7:115147-115159.

[6] B. Widrow et al. *Adaptive signal processing.* Prentince-Hall, 1986.

[7] E. Alonso et al. *A Machine Learning Framework for Pulse Detection During Out-of-Hospital Cardiac Arrest.* IEEE Access 2020;8:161031-41.

[8] G. James et al. *An Introduction to Statistical Learning: with Applications in R.* Springer, 2013.

[9] G. James et al. *An Introduction to Statistical Learning: with Applications in R.* Springer, 2013.

[10] C. Ding et al. *Minimum redundancy feature selection from microarray gene expression data.* Journal of Bioinformatics and Computational Bilogy 2005;3:185-205.

[11] S. D. Stearns et al. *On Selecting Features for Pattern Classifiers.* Third Int. Conf. on Pattern recognition 1976:71-75.
